# Preparing previously COVID-19-positive patients for elective surgery: a framework for preoperative evaluation

**DOI:** 10.1186/s13741-020-00172-2

**Published:** 2021-01-07

**Authors:** Naomi Bui, Mareli Coetzer, Katie J. Schenning, Avital Y. O’Glasser

**Affiliations:** 1 Department of Anesthesiology & Perioperative Medicine, Oregon Health & Science University, 3181 SW Sam Jackson Park Road, Portland, OR 97239 USA; 2 Department of Medicine, Oregon Health & Science University, 3181 SW Sam Jackson Park Road, CHH2 8008, Portland, OR 97239 USA

**Keywords:** Preoperative medicine, COVID-19, Elective surgery, Preoperative protocol

## Abstract

The preoperative evaluation and risk assessment has always been a critical aspect of safe surgical practice, and in the midst of the SARS-CoV-2 pandemic, it has become even more crucial to patient safety. Emerging data show that surgical procedures in patients who test positive for coronavirus disease (COVID) are associated with worse clinical outcomes and increased postoperative complications and mortality. In addition to personal protective equipment (PPE) management, isolation protocols, preoperative SARS-CoV-2 screening, and steps to ensure clinician safety, determining how to deem patients who have recovered from COVID-19 safe to proceed is an added challenge. We present a preoperative protocol for evaluation of previously COVID-positive patients for elective surgery.

## Introduction

As it evolves, the COVID-19 pandemic continues to impact nearly every aspect of clinical practice. With initial restrictions lifted and thousands of recovered COVID patients reentering the community, many of these patients are presenting for elective and non-urgent surgeries. However, there is limited evidence regarding the possible impact the disease may have on surgical risk and recovery. COVID-19 has revealed itself to cause multi-organ system disease, at times severe, with a protean list of clinical manifestations. Effects are not limited to systemic disease—prolonged hospital stays and post-viral syndromes are sequelae that should be taken into account when evaluating a patient for elective surgery. Previous studies have illustrated the potential for high morbidity and mortality in patients who undergo procedures while COVID positive, regardless of age or existing comorbidities (Nepogodiev et al. 2020; Doglietto et al. 2020). As of yet, there is little data and no recognized protocol for the preoperative evaluation of patients who were previously COVID positive but have since recovered.

Clinical manifestations of the disease include, but may not be limited to, respiratory failure, cardiomyopathy, arrhythmias, renal failure including the need for dialysis, liver function abnormalities, thromboembolic disease, endothelial dysfunction, and neurologic manifestations. However, therapeutic options are often limited to supportive therapy, and optimal anticoagulation therapy and duration of treatment in COVID-19 associated coagulopathy is still under investigation (Zhou et al. 2020). In addition, inpatient stays, including time spent on mechanical ventilation, can last for weeks to months. This can cause significant deconditioning that can contribute to frailty and poor outcomes (Belli et al. 2020). Some laboratory findings and biomarkers have been identified as predictors of disease severity, which may be useful in determining recovery and continued risk after acute COVID illness (Gao et al. 2020; Zhou et al. 2020; Zhang et al. 2020; Tang et al. 2020).

Furthermore, a new disorder known as “post-COVID syndrome,” describes patients that no longer have a viral infection, but still have remaining side effects of the illness including deconditioning and inflammation. Like many well-described viral illnesses, these post-viral symptoms can include profound fatigue, headaches, memory disturbances, difficulty with concentration, and depression. For example, chronic post-SARS side effects, characterized by fatigue, myalgia, weakness, depression, and poor sleep, were described in 2011 after the 2003 outbreak (Moldofsky and Patcai 2011). Similarly, there are reports of patients experiencing symptoms similar to chronic fatigue syndrome after mononucleosis infection, with memory and concentration deficits and profound exhaustion after exertion (Fugl and Andersen 2019). Many inflammatory syndromes and responses have been described in recovered COVID patients as well, with patients suffering from neurologic complications such as seizures, Guillain-Barre syndrome, and acute disseminated encephalomyelitis (Carroll et al. 2020; Chan et al. n.d.; Novi et al. 2020). Autoinflammatory diseases such as idiopathic thrombocytopenic purpura and autoimmune hemolytic anemia in adults, and pediatric inflammatory multisystemic syndrome, have developed in patients days to weeks after recovery (Zulfiqar et al. 2020; Galeotti and Bayry 2020; Lazarian et al. 2020). Ongoing recovery from COVID may be slow, and much is still unknown about long-term outcomes.

Given lack of evidence-based guidance on the recovery timeline, we draw parallels from the current literature on the importance of clinical recovery from a major medical event prior to proceeding with non-emergent surgery. For example, the recommended wait time after myocardial infarction is 8 weeks (Livhits et al. 2011) (with specific guidelines for patients who have undergone percutaneous coronary intervention). For stroke, the absolute minimum recovery time is 3 months, though risk continues to significantly decrease until 9 months (Mehdi et al. 2016; Jørgensen et al. 2014). Data regarding the appropriate timeline from upper respiratory infection (URI) recovery to surgery is limited for adult patients. In a study looking at postoperative complications in patients who had experienced a URI with fever requiring medical treatment in the month before surgery, postoperative complications, especially respiratory complications, were greater in patients who reported recent URI (Canet et al. 2008). However, given that there were no other time intervals studied, it is not possible to say whether the risk significantly decreases in patients who had a URI greater than one month prior surgery or if a shorter interval would increase morbidity. In addition, airway hyperreactivity has been found to last up to 6 weeks after URI (Aquilina et al. 1980).

Based on available evidence, and drawing analogies from other post-viral syndromes, we present a holistic framework for the preoperative evaluation of patients who previously had COVID-19. Factors in this assessment include the time period from clinical recovery from COVID-19, functional status, respiratory status, and imaging and laboratory data to determine a patient’s appropriateness for elective or non-urgent surgery. The protocol was vetted and approved by multidisciplinary stakeholders including surgical, anesthesiology, and hospital leadership. It outlines a multidisciplinary approach that also takes into account the psychosocial factors surrounding recovery from what can be a severe illness to achieve preoperative optimization and appropriately select when to proceed to the operating room.

## Protocol

In our protocol, all patients who have a history of a positive SARS-CoV-2 test that are scheduled for an elective surgery and/or procedure under general anesthesia must first undergo a comprehensive history and physical examination in our preoperative clinic. Minimum requirements prior to proceeding with surgery include complete resolution of COVID symptoms and adequate clinical recovery time. We chose a minimum recovery time of 4 weeks for patients who had an asymptomatic SARS-CoV-2 infection, and 6-8 weeks for symptomatic patients, acknowledging that there is currently little data on the timeframe of recovery. The history and physical portion emphasize details of the patient’s COVID course, signs and symptoms of potential subclinical COVID complications, determination of whether a patient has returned to their “pre-COVID” baseline health, a functional capacity assessment, and an ambulatory oxygen saturation measurement. Patients over the age of 65 or who required hospitalization for treatment of COVID regardless of age also undergo a frailty assessment using the Edmonton Frail Scale.

In addition to these basic requirements, objective testing is performed based on the severity of symptoms during COVID infection, the complexity of the surgical procedure, and the need for general anesthesia (Table 1). These tests serve to evaluate the patient’s cardiopulmonary function, coagulation status, markers of inflammation, and nutritional status. Given the aforementioned studies that show COVID can cause disturbances in these systems, an abnormal value may signal incomplete resolution of the disease, possibly increasing risk of intra- or postoperative complications. We elected not to perform arterial blood gasses (ABG) as the complete metabolic panel (CMP) and ambulatory oxygen measurement provide similar data without incurring the risk of hematoma, nerve injury, or arterial injury. Though some studies have found a correlation between severity of illness in recovered COVID-19 patients and decreased diffusing capacity for carbon monoxide (DLCO) (Zhao et al. 2020; Mo et al. 2020), as of yet, there is unclear clinical significance with regard to perioperative outcomes. Therefore, we have currently excluded pulmonary function tests (PFT) from our protocol as their routine use is not recommended in non-thoracic surgeries.

**Table 1:** Protocol for the preoperative objective assessment of COVID-19 survivors, stratified based on nature of planned surgery and degree of index illness

Step/test	Minor procedures and/or without general anesthesia		Major procedures	
	Asymptomatic	Symptomatic	Asymptomatic	Symptomatic
CXR	No—if pulmonary exam and O_2_ sat normal	No—if pulmonary exam and O_2_ sat normal	Yes	Yes
EKG	Yes	Yes	Yes	Yes
Echo	No—if cardiac exam and vitals normal	No—if cardiac exam, NT-pro-BNP, and vitals normal	No—if cardiac exam, NT-pro-BNP, and vitals normal	Determined by H&P
CMP	Yes	Yes	Yes	Yes
CBC, with diff	Yes	Yes	Yes	Yes
PTT	No	Consider based on severity of illness	Yes	Yes
D-dimer	No	Yes	Yes	Yes
Fibrinogen	No	Consider based on severity of illness	Yes	Yes
NT-pro-BNP	No	Yes	Yes	Yes
LDH, ferritin, prealbumin	No	Consider based on severity of illness	No	Consider based on severity of illness

After this assessment, patients with normal results may proceed after the minimum wait period. However, any significant abnormalities would trigger a multidisciplinary discussion and consultations with other specialties as appropriate. In the case of non-elective time-sensitive surgeries, the protocol will be completed as thoroughly as possible. The patient would then have detailed risk-benefit counseling and/or goals of care discussions before proceeding to surgery.

Our protocol was approved and introduced to the institution mid-August 2020. Approaching the 4-month mark, we have evaluated approximately 40 patients, with a spectrum of illness ranging from asymptomatic to severe infection. We are not aware of any patients who had their surgeries delayed/canceled due to abnormalities in their evaluations. We are aware of only two patients who had surgery delayed due to time requirements; we credit proactive partnership with referring surgeons to schedule surgery and preoperative evaluation when the time-based requirements had already been met. Next phases include the expansion of our protocol to patients with past COVID-19 infection presenting for procedural sedation for minor procedures.

## Conclusion

As millions of people in the USA have recovered from COVID and are presenting for elective and non-urgent surgeries, we feel it is appropriate to set a standard for preoperative evaluation given the high risk of complications and high degree of clinical uncertainty. As we see a growing number of asymptomatic patients who incidentally test positive during their preoperative screening, this protocol also provides a clear framework to follow for this patient population. We know that patients who undergo surgery with active COVID infection fare significantly worse, but the timeline for recovery remains nebulous. The onus is on us to view contracting and recovering from COVID as we do any other serious medical event, with appropriate presurgical evaluation to prepare and optimize these patients for elective surgery. We hope that our single-center experience will add to the growing body of literature about this clinical challenge.

## Data Availability

Data sharing is not applicable to this article as no datasets were generated or analyzed during the current study.

## Abbreviations

URI: Upper respiratory infection
CXR: Chest x-ray
EKG: Electrocardiogram
ECHO: Echocardiogram
CMP: Complete metabolic panel
CBC with Diff: Complete blood count with differential
PTT: Partial thromboplastin time
LDH: Lactate dehydrogenase
nt-pro-BNP: N-terminal pro brain natriuretic peptide
